# Force-Dependent Folding Kinetics of Single Molecules with Multiple Intermediates and Pathways

**DOI:** 10.1021/acs.jpclett.1c03521

**Published:** 2022-01-24

**Authors:** Marc Rico-Pasto, Anna Alemany, Felix Ritort

**Affiliations:** † Small Biosystems Lab, Condensed Matter Physics Department, University of Barcelona, C/Martí i Franqués 1, Barcelona, 08028, Spain; ‡ Department of Anatomy and Embryology, Leiden University Medical Center, Leiden, 2333ZC, The Netherlands

## Abstract

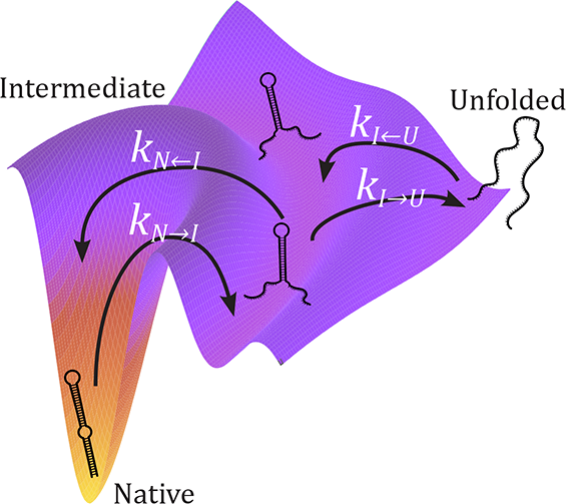

Most single-molecule studies derive the kinetic rates of native, intermediate, and unfolded states from equilibrium hopping experiments. Here, we apply the Kramers kinetic diffusive model to derive the force-dependent kinetic rates of intermediate states from nonequilibrium pulling experiments. From the kinetic rates, we also extract the force-dependent kinetic barriers and the equilibrium folding energies. We apply our method to DNA hairpins with multiple folding pathways and intermediates. The experimental results agree with theoretical predictions. Furthermore, the proposed nonequilibrium single-molecule approach permits us to characterize kinetic and thermodynamic properties of native, unfolded, and intermediate states that cannot be derived from equilibrium hopping experiments.

Some nucleic acids and proteins require intermediate or partially folded configurations to perform their biological function. For example, RNA riboswitches are regulatory molecules that induce or repress gene transcription depending on their conformation;^1^ RNA thermometers act like lockers whose ribosomal binding site becomes accessible only at high temperatures when they partially unfold;^2,3^ and proteins fold into the native structure by forming intermediate folding units (foldons).^4,5^ Therefore, a quantitative characterization of the dynamical formation of intermediates is a critical step toward the elucidation of many molecular processes. Accordingly, it is of high interest to develop accurate tools to investigate the thermodynamics and kinetics of partially folded domains occurring in biomolecules.

Single-molecule methods provide an ideal ground to experimentally address these questions since they allow us to sample transient molecular states with high temporal (∼ms) and spatial (∼nm) resolution.^6^ In particular, atomic force microscopy,^7,8^ magnetic^9,10^ and optical tweezers,^11−13^ permit us to pull on individual molecules and to monitor unfolding/folding reactions from the recorded changes in extension, the reaction coordinate in these experiments.^14,15^

Single-molecule techniques have been used to characterize intermediates in a wide variety of molecular systems, from protein folding^16−18^ and binding metal–metalloproteins^19−21^ to RNA and DNA folding,^22−24^ G-quadruplex DNA formation,^25,26^ DNA duplexes formation with base-pair mismatches,^27^ and synthetic molecular foldamers and shuttles.^28,29^ Moreover, upon misfolding, molecular intermediates have also been shown to play a role, for example, in neuronal calcium sensors.^30^

Dynamic force spectroscopy studies are often performed in equilibrium conditions, for example, in hopping experiments.^31−36^ There, the control parameter (e.g., trap position in optical tweezers, Figure 1a) is kept fixed as the molecule executes thermally driven transitions between different molecular states. In such experiments, the unfolding and folding kinetics are derived from the average lifetime of each state.^37,38^ However, equilibrium experiments are strongly limited by the height of the kinetic barrier, *B*, mediating transitions between contiguous states along the molecular free energy landscape (mFEL) (Figure 1b). A too high kinetic barrier (*B* ≫ *k*_B_*T*, *k*_B_ being the Boltzmann constant and *T* the temperature) prevents molecular transitions over measurable time scales, leading to inefficient sampling of the conformational space. Instead, nonequilibrium experiments facilitate transitions over large kinetic barriers, providing an alternative and efficient way to sample the mFEL. Examples are jump experiments in which a system is driven to a new state by suddenly changing an external parameter (such as temperature, force, pH, etc.), and the system’s relaxation is monitored.^39−41^

**Figure 1:**
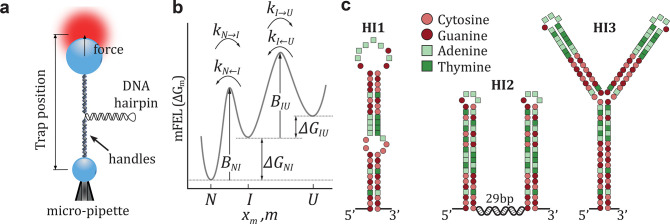
Experimental setup and DNA sequences. (a) Schematics of a pulling experiment with optical tweezers. The DNA hairpin is tethered between two beads using double-stranded DNA handles. One bead is fixed by air suction on the tip of a micropipette while the other is controlled by the optical trap. (b) Illustrative mFEL with an intermediate state. The unfolding and folding kinetic rates and barriers are indicated. (c) Sequences of the studied DNA molecules.

Two widely used phenomenological approaches to extract equilibrium information from pulling experiments are the Bell-Evans (BE)^42−44^ and the kinetic diffusive (KD) models. The BE model describes mechanically induced folding/unfolding transitions as thermally activated processes over a transition state energy barrier. The BE model assumes that, for a fixed transition state position, the height of the kinetic barrier decreases linearly with the applied force, *B* = *B*_0_ – *fx*^†^ (*x*^†^ being the distance from the departure state to the transition state). This assumption is relaxed in the KD model, which considers the folding reaction as a diffusive process in a one-dimensional force-dependent mFEL (Figure 1b). While the BE model only considers the height and position of the transition state, the full description of the mFEL in the KD model requires the knowledge of all the partially folded intermediate conformations. The advantage of the KD is the high predictive power. The same experimental data can be readily employed to extract additional information about the mFEL without the need to adopt the assumptions of the BE model. The KD model has been applied to study the folding kinetics of two-state nucleic acid hairpins and proteins.^33,35,45,46^

A useful method based on the KD model is the Continuous Effective Barrier Approach (CEBA). Originally introduced to study RNA hairpins,^47^ it was later applied to extract the elastic properties of short RNA hairpins at different ionic conditions,^48^ the thermodynamic and kinetic properties of protein Barnase,^49^ and DNA hairpins with different mechanical fragilities.^50^ In CEBA, the force-dependent effective barrier between the native (*N*) and the unfolded state (*U*), *B*_*NU*_(*f*), is derived by imposing detailed balance between the unfolding (*k*_*N*→*U*_(*f*)) and folding (*k*_*N*←*U*_(*f*)) kinetic rates:
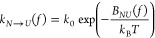
1a
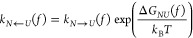
1bHere *k*_0_ is the attempt rate and Δ*G*_*NU*_(*f*) is the folding free energy at force *f*,
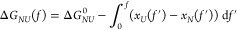
2where Δ*G*_*NU*_^0^ is the folding free energy difference between *N* and *U* at zero force, and – ∫_0_^*f*^*x*_*U*(*N*)_(*f*′) d*f*′ the free energy decrease upon stretching the molecule in state *U*(*N*) at force *f*. The elastic response of *U* and *N* are modeled using the Worm-Like Chain and Freely-Jointed Chain models.^51^Equations 1a, 1b are conveniently rewritten as
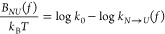
3a
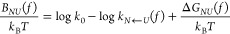
3bTherefore, by equating eq 3a and eq 3b, the difference between −log *k*_*N*→*U*_(*f*) and −log *k*_*N* ← *U*_(*f*) – (1/*k*_B_*T*) ∫_0_^*f*^ (*x*_*U*_(*f*′) – *x*_*N*_(*f*′)) d*f*′ equals Δ*G*_*NU*_^0^ (c.f., eq 2). This permits us to derive the folding free energy *ΔG*_*NU*_^0^ if the elastic response (*x*_*U*_ (*f*) – *x*_*N*_ (*f*)) is known. Moreover, we extract *k*_0_ by comparing the experimental profile of *B*_*NU*_ (*f*) – log *k*_0_ with the theoretically predicted *B*_*NU*_ (*f*) by the KD model.^49,50^ For a DNA hairpin, the latter is given by^52−54^ (a derivation can be found in section S1 of Supporting Information)
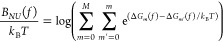
4where the double sum runs over all hairpin configurations, labeled by *m* and *m*′, and *M* is the total number of base pairs (bp).

CEBA has been mostly applied to molecules with two distinct molecular states, that is, *N* and *U*, separated by a kinetic barrier.^47−50^ Here, we extend CEBA (hereafter referred to as eCEBA) to investigate molecular reactions involving intermediate kinetic states from nonequilibrium pulling experiments. We use optical tweezers to pull DNA hairpins with one, two, and three intermediates (Figure 1a). The existing knowledge about DNA thermodynamics^11,55−57^ allows us to accurately predict the force-dependent kinetic barriers of arbitrary sequences, facilitating the comparison between theory and experiments. The chosen examples cover situations often encountered in macro-molecular folding.

## A Single Intermediate and Folding Pathway

**Figure 2:**
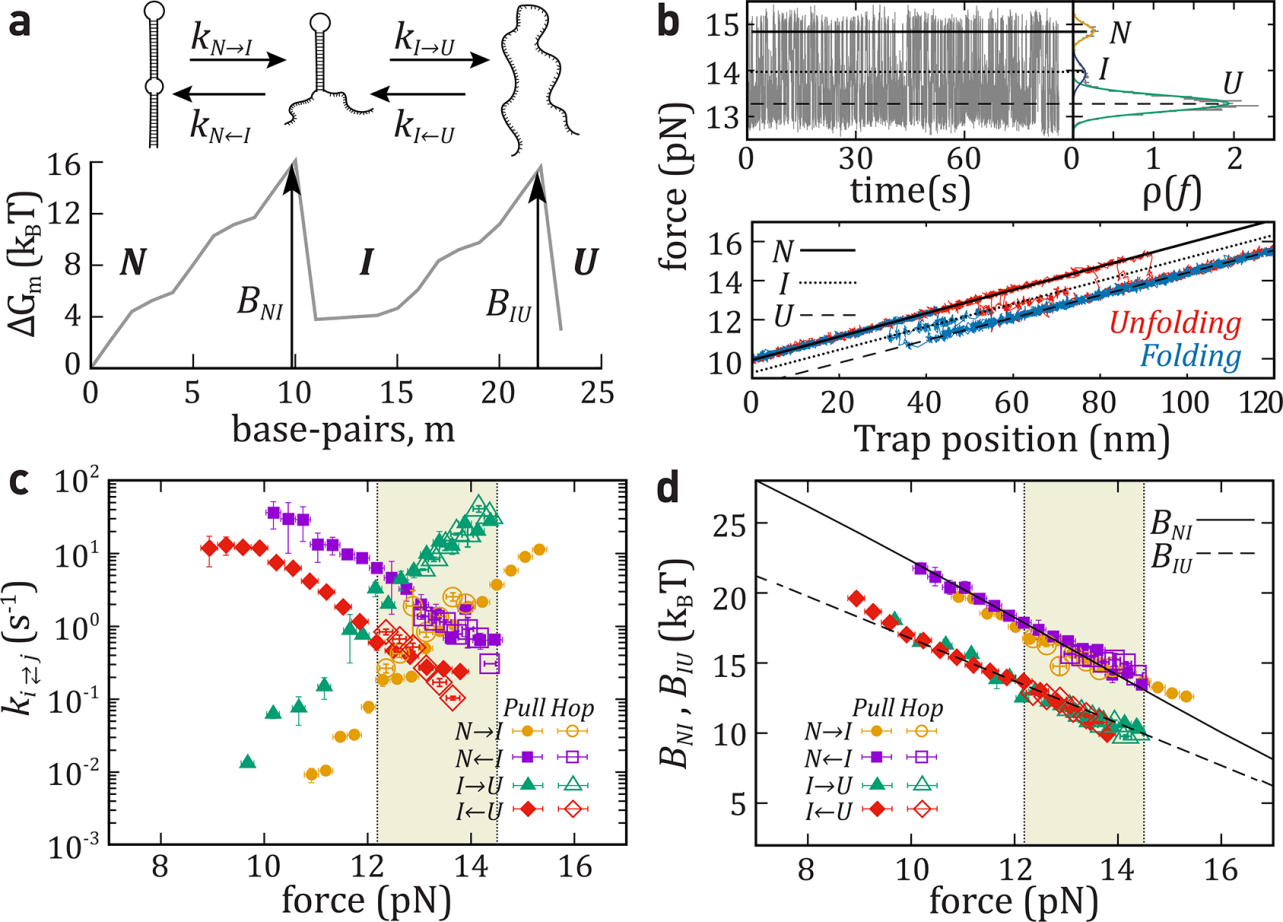
Unfolding–folding kinetics of a single intermediate (hairpin HI1). (a) Top: Schematic unfolding and folding pathway for HI1. Bottom: mFEL (Δ*G*_*m*_) as a function of the number of unfolded base-pairs (*m*) at 15pN. The barriers, *B*_*NI*_ and *B*_*IU*_, are highlighted (black arrows). (b) Top-left: Force versus time trace measured for HI1. Top-right: Histogram of the force signal used to recognize the three states (*N*, *I*, and *U*). Bottom: Five unfolding (red) and folding (blue) FDCs (pulling speed equals 100 nm/s, each trajectory taking ∼2 s). Force branches for states *N* (black solid line), *U* (black dashed line), and *I* (dotted line). (c) Kinetic rates of unfolding, *k*_*N*→*I*_ (yellow circles) and *k*_*I*→*U*_ (green triangles); and folding, *k*_*N* ← *I*_ (purple square) and *k*_*I* ← *U*_ (red diamond). Kinetic rates derived from nonequilibrium (solid symbols) and equilibrium (empty symbols) experiments. (d) Barriers mediating transitions between *N* and *I* (black solid line) and between *I* and *U* (black dashed line) as predicted from eqs 9a,9b compared with the experimental results (symbols). Shaded regions in panels c and d show the range of forces where kinetic rates can be measured in equilibrium hopping experiments. Results are the average over four different molecules, and the error bars correspond to the statistical errors.

The first DNA hairpin (denoted as HI1) has an internal loop in the stem (Figure 1c) that stabilizes an intermediate (*I*) upon folding/unfolding, as shown in the theoretical prediction of the mFEL^55−58^ calculated at 15pN (Figure 2a). Partial folding and unfolding connecting states *N*, *I*, and *U* can be observed as sudden drops and rises of force, respectively, in hopping (equilibrium) and pulling (nonequilibrium) experiments (Figure 2b). In hopping experiments, the molecule is held at a fixed trap position (distance), and each observed level of force corresponds to a different state (Figure 2b, top). In pulling experiments, the trap position is moved back and forth at a constant speed and the molecule is repeatedly folded and unfolded. The different force branches observed in the force–distance curves (FDCs) arise from the elastic response of the hairpin in each state (black lines in Figure 2b, bottom). Let us note that fast hopping events are missed in force feedback protocols with optical tweezers, underestimating the kinetic rates.^38^ Then, a proper comparison between hopping and pulling should be done in the same experimental condition (either controlling force or distance).

In hopping experiments, the kinetic rates *k*_*N*→*I*_(*f*), *k*_*I*→*U*_(*f*), *k*_*N*←*I*_(*f*), and *k*_*I*←*U*_(*f*) are derived from the lifetime of each state (empty symbols in Figure 2c). In pulling experiments, we determine them from the survival probabilities of each state along the unfolding and folding FDCs. The methodology to determine the survival probabilities for molecules with an arbitrary number of intermediates is very general. For the single intermediate case it is as follows. First, we set a threshold force, *f*_th_, and measure the first rupture (formation) event taking place at a force above (below) *f*_th_ for each unfolding (folding) trajectory. Next, we classify the force events as *f*_→_^*i*^ and *f*_←_^*i*^, where *i* = *N*, *I* or *U* indicates the molecular state at *f*_th_ and the arrow indicates the direction of the FDCs: unfolding (→) or folding (←). Note that for *i* = *I*, *f*_→_^*I*^ and *f*_←_^*I*^ comprise both rupture and formation events indistinguishably, while *f*_→_^*N*^ and *f*_←_^*N*^ only contain rupture events and *f*_→_^*U*^ and *f*_←_^*U*^ only contain formation events. From *f*_→_^*i*^, *f*_←_^*i*^, we calculate the force-dependent survival probabilities conditioned to *f*_th_ along the unfolding (*P*_→_^*N*^(*f* | *f*_th_), *P*_→_^*I*^(*f* | *f*_th_), *P*_→_^*U*^(*f* | *f*_th_)) and folding (*P*_←_^*N*^(*f* | *f*_th_), *P*_←_^*I*^(*f* | *f*_th_), *P*_←_^*U*^(*f* | *f*_th_)) trajectories:
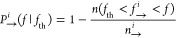
5a
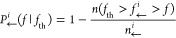
5bwhere *n*(*f*_th_ < *f*_→_^*i*^ < *f*) (*n*(*f*_th_ > *f*_←_^*i*^ > *f*)) denotes the number of events during unfolding (folding) leaving state *i* for the first time between *f*_th_ and *f*, and *n*_→_^*i*^ (*n*_←_^*i*^) is the total number of trajectories with state *i* observed at *f*_th_. Note that by construction, *P*_→(←)_^*i*^ (*f*_th_ | *f*_th_) = 1. By repeating the analysis for different values of *f*_th_, we reconstruct *P*_→_^*i*^ (*f*|*f*_th_) and *P*_←_^*i*^ (*f*|*f*_th_) for different values of *f*_th_ and *f*. The survival probabilities satisfy the following master equations:
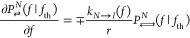
6a
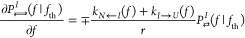
6b
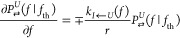
6cwhere *k*_*N*→*I*_(*f*), *k*_*N*←*I*_(*f*), *k*_*I*→*U*_(*f*), and *k*_*I*←*U*_(*f*) are the kinetic rates between the states, and *r* = |d*f*/d*t*| is the constant loading rate. The −(+) sign in the right hand side denotes the unfolding (folding) processes.

**Figure 3:**
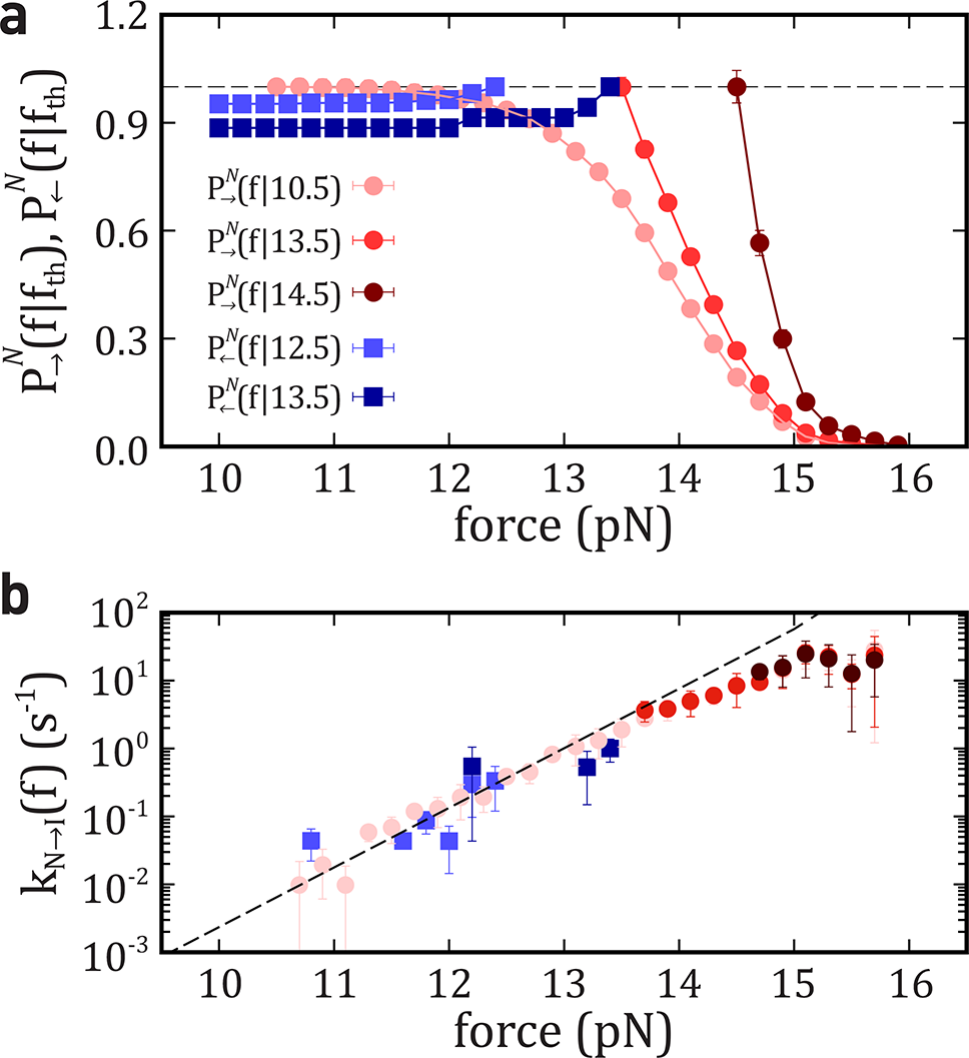
Survival probability of *N* and derived kinetic rate *N* → *I*. (a) Survival probability of *N* in the unfolding (red circles) and folding (blue squares) processes. (b) Derived *k*_*N*→*I*_(*f*) using eq 6a. Data fall on the same line. Error bars are the statistical errors over five molecules. The black dashed line is the prediction by the KD model.

For a Markovian system, eqs 5a and 6c give estimates of *k*_*N*⇄*I*_, *k*_*I*⇄*U*_ that are independent of the value *f*_th_ and the process → (←). Therefore, by merging results obtained at different *f*_th_ and → (←) we optimize the available data improving kinetic rates estimates. In Figure 3a, we show *P*_⇄_^*N*^(*f*|*f*_th_) for three values of *f*_th_ for → and two *f*_th_ values for ← processes. The corresponding *k*_*N*→*I*_(*f*) values derived from eq 6a are compatible with each other (Figure 3b).

A similar procedure is used to determine *k*_*I*←*U*_(*f*). To decouple *k*_*N*←*I*_(*f*) from *k*_*I*→*U*_(*f*) in eq 6b, we use the following relation:
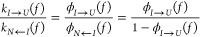
7where ϕ_*I*→*U*_(*f*) and ϕ_*N*←*I*_(*f*) are the fraction of transitions leaving *I* toward *U* and *I* toward *N*, respectively, at force *f* (ϕ_*I*→*U*_(*f*) + ϕ_*N*←*I*_(*f*) = 1). These fractions are experimentally measured on a force window Δ*f* = 0.1 pN.

Figure 2c shows a good agreement between kinetic rates recovered from hopping (empty symbols) and pulling experiments (solid symbols). Notably, the force range where transitions are observed in hopping (highlighted in yellow) is narrower compared to that from pulling experiments. This shows that nonequilibrium pulling experiments provide kinetic rates over a wider force range.

Next, we use eCEBA to determine the effective barriers *B*_*NU*_(*f*) and *B*_*IU*_(*f*) from the kinetic rates by generalizing eqs 3a and 3b to states *N*, *I*, *U*:
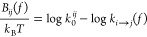
8a
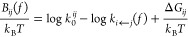
8bwhere *i*,*j* ∈{*N*,*I*,*U*}, *k*_*i*→*j*_, *k*_*i*←*j*_ is the unfolding and folding kinetic rates between *i* and *j*, and *k*_0_^*ij*^ is the attempt rate. Δ*G*_*ij*_ equals Δ*G*_*ij*_^0^ – ∫_0_^*f*^(*x*_*j*_(*f*′) – *x*_*i*_(*f*′)) d*f*′, where ∫_0_^*f*^*x*_*k*_(*f*′) d*f*′ is the energy cost to stretch state *k* up to force *f*, and Δ*G*_*ij*_^0^ is the folding free energy difference at zero force between states *i* and *j*.

By imposing continuity between the two expressions for *B*_*ij*_(*f*)/*k*_B_*T* – log *k*_0_^*ij*^ in eqs 8a, 8b, we derive Δ*G*_*ij*_^0^ for *ij* = *NI* and *ij* = *IU*. We also estimate the attempt frequencies *k*_0_^*ij*^ by matching the experimental results for *B*_*ij*_(*f*) with the theoretical Kramers prediction, calculated as^52^
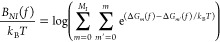
9a
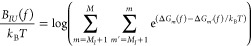
9bwhere the double sum runs over all hairpin configurations, labeled by *m* and *m*′, *M*_*I*_ being the number of unzipped base-pairs (bp) at *I*, and *M* being the total number of bp.

The resulting barriers are shown in Figure 2d (solid symbol, pulling; empty symbol, hopping), while the extracted values for Δ*G*_*ij*_^0^ and *k*_0_^*ij*^ are summarized in Table 1. We find good agreement with theoretical predictions.

**Table 1:** Folding Free Energies and Kinetic Attempt Rates for the Three Studied Hairpins^a^

			Δ*G*_*i*,*j*_^0^ (*k*_B_*T*)	
	*i*, *j*	*k*_0_^*ij*^ (s^–1^)	exp.	pred.
HI1	*N*, *I*	(5 ± 1) × 10^7^	30 ± 2	30 ± 1
	*I*, *U*	(7 ± 1) × 10^6^	27 ± 3	28 ± 1
	*N*, *I*	(5 ± 1) × 10^7^	31 ± 3	30 ± 1
	*I*, *U*	(6 ± 1) × 10^6^	28 ± 4	28 ± 1
HI2	*N*, *I*	(5 ± 1) × 10^5^	54 ± 2	52 ± 2
	*I*, *U*	(2 ± 1) × 10^6^	51 ± 1	55 ± 2
HI3	*N*, *I*_1_	(6 ± 1) × 10^5^	57 ± 4	52 ± 2
	*I*_1_, *I*_2_^′^	(2 ± 1) × 10^6^	38 ± 3	41 ± 2
	*I*_2_^′^, *U*	(9 ± 2) × 10^5^	39 ± 2	40 ± 2

a The results of molecule HI1 in the top (bottom) rows correspond to pulling (hopping) experiments. The error bars for the experimental values correspond to the statistical error considering all studied molecules, while the error bar in the Mfold prediction corresponds to the standard error considering several experimental values.

## A Doubly Degenerate Intermediate and Two Folding Pathways

**Figure 4:**
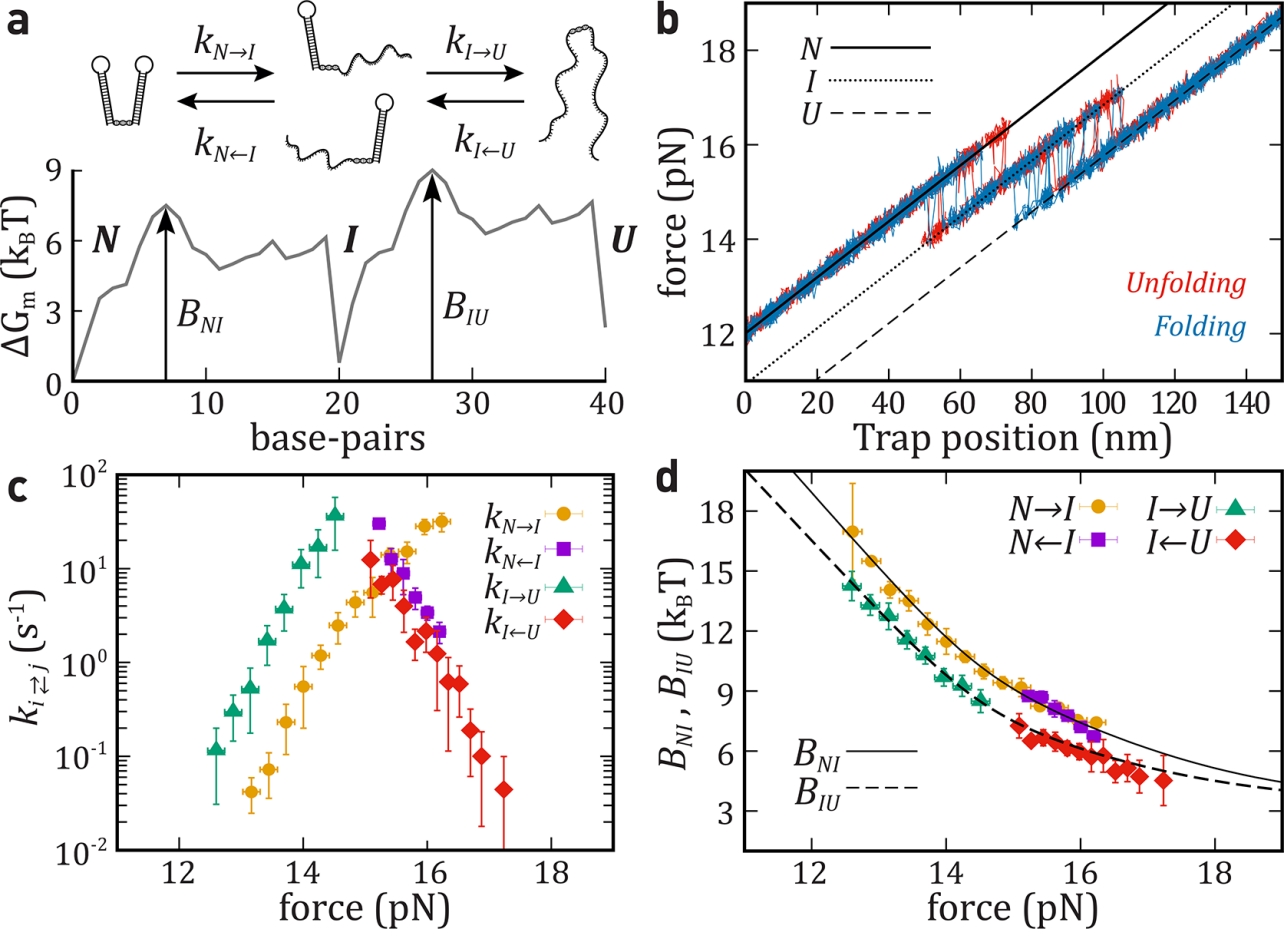
Unfolding–folding kinetics of a doubly degenerate intermediate (hairpin HI2). (a) Top: Schematic unfolding and folding pathways for HI2 that has two degenerate intermediates (both hairpins have the same sequence) depending which of the two hairpins is unfolded. Bottom: mFEL (Δ*G*_*m*_) as a function of the number of unfolded base-pairs (*m*) at 15pN. The barriers, *B*_*NI*_ and *B*_*IU*_, are highlighted (black arrows). (b) Five unfolding (red) and folding (blue) FDCs (pulling speed equals 100 nm/s, each trajectory taking ∼2 s). Force branches for states *N* (black solid line), *U* (black dashed line), and *I* (dotted line). (c) Kinetic rates of unfolding, *k*_*N*→*I*_ (yellow circle) and *k*_*I*→*U*_ (green triangle); and folding, *k*_*N*←*I*_ (purple square) and *k*_*I*←*U*_ (red diamond). (d) Barriers mediating transitions between *N* and *I* (black solid line) and between *I* and *U* (black dashed line) predicted from eqs 9a,9b) and compared with the experimental results (symbols). The results shown in panels c and d are the average over four different molecules, and the error bars correspond to the statistical errors.

Next, we designed hairpin HI2, which contains two identical DNA hairpins serially connected and separated by a short (29bp) double-stranded DNA segment (Figure 1c). The native hairpin *N* can unfold via two different pathways, each characterized by an intermediate corresponding to the unfolding of one of the two hairpins (Figure 4a). However, as both hairpins are identical, they cannot be experimentally distinguished. Therefore, we define a global intermediate *I* comprising the two intermediates.

The mFEL of HI2 is defined as the potential of mean force where a given number *m* of open bps (0 ≤ *m* ≤ 40) is distributed among the two hairpins. The mFEL shows a single intermediate at *m* = 20 (Figure 4a-bottom), where one hairpin is folded, and the other is unfolded.

In Figure 4b we show unfolding (red) and folding (blue) FDCs. Like for HI1 there are three force branches for states *N*, *I*, and *U* (black lines). We use eCEBA to determine the force-dependent kinetic rates (Figure 4c) and the effective barriers *B*_*NI*_ and *B*_*IU*_ mediating transitions between the three states (Figure 4d). Results for the folding free energies and attempt rates are shown in Table 1 (middle). Note that, although both hairpins are identical, the barriers for *N* ⇄ *I* and *I* ⇄ *U* are different (Figure 3d).

## Three Intermediates and Two Folding Pathways

**Figure 5:**
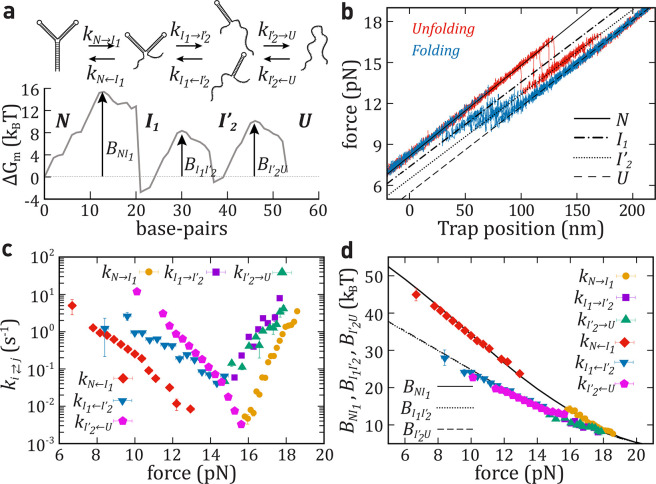
Unfolding–folding kinetics of a triple intermediate (hairpin HI3). (a) Top: Schematic unfolding and folding pathways for HI3. Bottom: mFEL (Δ*G*_*m*_) as a function of the number of unfolded base-pairs (*m*) at 15pN. The barriers, *B*_*NI*_1__, *B*_*I*_1_,*I*_2_^′^_, and *B*_*I*_2_^′^*U*_, are highlighted (black arrows). (b) Five unfolding (red) and folding (blue) FDCs (pulling speed equals 100 nm/s, each trajectory taking ∼2 s). Force branches for states *N* (black solid line), *U* (black dashed line), *I*_1_ (dotted-line line), and *I*_2_ (dotted line). (c) Kinetic rates of unfolding: *k*_*N*→*I*_1__ (yellow circle), *k*_*I*_1_→*I*_2_^′^_ (purple square), and *k*_*I*_2_^′^→*U*_ (green triangle); and folding: *k*_*N*←*I*_1__ (red diamond), *k*_*I*_1_←*I*_2_^′^_ (blue down-pointing triangle), and *k*_*I*_2_^′^←*U*_ (pink pentagon). (d) Barriers mediating transitions between *N* and *I*_1_ (black solid line), between *I*_1_ and *I*_2_^′^ (black dotted line), and between *I*_2_^′^ and *U* (black dashed line) predicted from eqs 9a and 9b extended to one more intermediate and compared with the experimental results (symbols). The results shown in panels c and d are the average over five different molecules, and the error bars correspond to the statistical errors.

The last studied molecule (HI3) is a DNA three-way junction (Figure 1c). For the first intermediate, *I*_1_, the 20 bps of the main stem (before the junction) are unzipped. Further unzipping of HI3 distributes open bps between the two upper arms of HI3. The calculated mFEL (Figure 5a, bottom) shows two additional intermediates, each for the unfolding of one arm. Therefore, HI3 can take two different pathways to unfold starting from *I*_1_: *I*_1_ → *I*_2_ → *U* or *I*_1_ → *I*_3_ → *U* depending on which arm is opened first. Since we cannot distinguish between *I*_2_ and *I*_3_ from the FDCs (Figure 5b), we studied the unfolding and folding pathway as *N* ⇆ *I*_1_ ⇆ *I*_2_^′^ ⇆ *U*. Here, *I*_2_^′^ comprises *I*_2_ and *I*_3_: *I*_2_^′^ = *I*_2_ ∪ *I*_3_.

In Figure 5c we show the six kinetic rates of HI3. From eqs 8a and 8b, we derive the effective barrier, the free energy difference, and attempt rates for *N* ⇆ *I*_1_, *I*_1_ ⇆ *I*_2_^′^, and *I*_2_^′^ ⇆ *U* (Table 1). In Figure 5d we show *B*_*NI*_1__(*f*), *B*_*I*_1_*I*_2_^′^_(*f*), and *B*_*I*_2_^′^*U*_(*f*) together with the theoretical prediction from eqs 9a and 9b extended to include a second intermediate. Due to the sequence similarity between the two arms in the three-way junction, the barriers for *I*_2_^′^ ⇆ *U* and *I*_1_ ⇆ *I*_2_^′^ are nearly equal.

## Discussion

In the present work, we used nonequilibrium pulling experiments to determine the force-dependent unfolding/folding kinetic rates for DNA hairpins with three different kinds of intermediates (Figure 1c): a hairpin with an inner-loop and a single intermediate (HI1); a two-hairpin structure with a doubly degenerate intermediate (HI2); and a three-way junction with three intermediates (HI3). For hairpin HI1, we also derived the kinetic rates from equilibrium hopping experiments. We showed that pulling experiments recover kinetic rates at forces where intermediates cannot be sampled in equilibrium conditions. In general, the force gap between the unfolding and folding forces facilitates reconstructing the kinetic barrier *B*_*ij*_(*f*) in a larger force range.^49^ Further extension of the range of forces where kinetic rates are measured might be achieved by increasing (decreasing) the loading (unloading) rate during the unfolding (folding) process. The simplicity of the BE model^42−44^ makes it a preferred model to fit the kinetic rates. Here we exploited eCEBA^47−50^ to measure the force-dependent kinetic barriers, *B*_*ij*_, and the free energy differences, *ΔG*_*ij*_, between different states. Our results showed good agreement between the experimental values and the predictions based on the nearest neighbor model (Table 1). The folding free-energy values per bp of the nearest-neighbor model used in the comparison are obtained from the Mfold. The latter uses energy parameters derived from temperature melting data collected in calorimetry (bulk) experiments.^55,56^ The good agreement between the measured force-dependent kinetic barriers and the KD model prediction allowed us to estimate values for attempt rates for native, intermediate, and unfolded states. Attempt rates are important for molecular dynamic simulations, for which time scales need to be set properly. In general, the KD model has more predictive power than the BE model which assumes a single kinetic barrier between states. In contrast, in the KD model, folding is a diffusive process in a one-dimensional mFEL with many intermediate configurations. In principle, eq 4 (two-states) and eq 9a, (9b) (three states) might be inverted (by discretizing the force range) to derive the energy set, Δ*G*_*m*_^0^, directly from the measured *B*_*ij*_(*f*).

Notice that HI2 and HI3 were designed to have degenerated and indistinguishable folding intermediates. For nondegenerate and distinguishable intermediates, the analysis of the respective folding pathways follows the same steps as we did for HI1 (Figure 2). However, for nondegenerate and indistinguishable intermediates, dynamics might not be Markovian and the KD model (c.f., eq 4) should be revisited.

In cases where the mFEL is not known (e.g., in tertiary RNAs and proteins), eq 4 is inapplicable. However, one can still reconstruct the kinetic barrier by using eq 1a from the measured *k*_*N*→*U*_(*f*) and the knowledge of *k*_0_. The latter can be obtained from the extrapolated value of *B*_*NU*_(*f*) to zero force which is approximately equal to Δ*G*_*NU*_^0^, . Determining *k*_*N*→*U*_(*f* = 0) requires reconstructing the kinetic rates at low forces. In section S2 of Supporting Information, we describe the procedure used to reconstruct the kinetic rates and barriers down to zero force. Results are tested for hairpin HI1 finding results in agreement with those summarized in Table 1.

Future studies might address kinetic barrier measurements at different temperatures^38,59−61^ to separate the enthalpic and entropic contributions. These studies might be applied to other non-native states, for example, misfolded structures. eCEBA might also find applications to unravel the kinetic role of complex molecules, such as chaperons and other enzymes that facilitate molecular folding/unfolding reactions, and ligand binding. The possibility of characterizing changes in the kinetic barrier’s height as a function of force under different conditions (e.g., crowding, binding agents, temperature, ionic strength) will permit us to understand how molecular machines in cells respond to external signals and perturbations.

## Supporting Information Available

Detailed derivation of the kinetic barrier in the KD model; reconstructing the kinetic rates for complex molecules (PDF)
