# Supplementary material for: Force-Dependent Folding Kinetics of Single Molecules with Multiple Intermediates and Pathways
Source: J Phys Chem Lett. 2022 Jan 24;13(4):1025–32. doi: 10.1021/acs.jpclett.1c03521 (PMC9882750; doi:10.1021/acs.jpclett.1c03521)
Supplement: Supplementary file 1 — jz1c03521_si_001.pdf [file jz1c03521_si_001.pdf]

Supplementary Information: Force-Dependent Folding Kinetics of Single Molecules with Multiple Intermediates and Pathways

Marc Rico-Pasto,† Anna Alemany,‡ and Felix Ritort*,†

† *Small Biosystems Lab, Condensed Matter Physics Department, University of Barcelona, C/Martí i Franqués 1, 08028 Barcelona, Spain*

‡*Department of Anatomy and Embryology, Leiden University Medical Center, Leiden, The Netherlands*

E-mail: ritort@ub.edu

S1 Derivation of the kinetic barrier in the KD model

Let us consider the system presented in Fig. S1, where a Brownian particle diffuses in one dimensional potential of mean force $V(x)$. The time evolution of the probability density function $p(x,t)$ to find the particle at position x at time t follows the Fokker-Planck equation,[1,2]

$$\frac{\partial p(x,t)}{\partial t} = D\frac{\partial}{\partial x}\left[\frac{\partial}{\partial x} + \frac{1}{k_B T}\frac{dV(x)}{dx}\right]p(x,t) \tag{S1}$$

where $D = k_B T/\gamma$ is the diffusion coefficient, γ is the friction coefficient, k_B is the Boltzmann constant, and T is the temperature.

By considering that the particle at time $t=0$ is located in a region $\mathcal{R}=[a,b]$ at position $x_0 \in \mathcal{R}$, then $p(x,0)=\delta(x-x_0)$. Considering the survival probability defined as the proba-

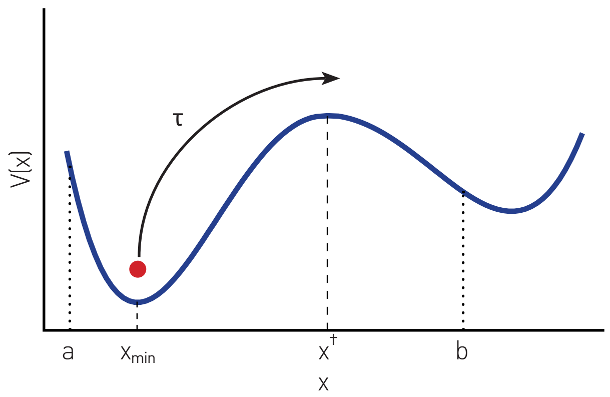

Figure S1: **Brownian particle in a double well potential.** The minim and maximum values of $V(x)$ are located at x_{min} and $x^{\dagger}$, respectively. Positions a and b are used to model the absorbing and reflecting boundaries of the dynamics of the particle (red dot).

bility of the Brownian particle to remain inside $\mathcal{R}$ at time t, $\mathcal{S}_{x_0}(t,\mathcal{R}) = \int_{x\in\mathcal{R}} p(x,t)dx$, it is possible to determine the density function of the survival time, which is equal to $-\frac{\partial \mathcal{S}_{x_0}(t,\mathcal{R})}{\partial t}$. The mean first passage time τ is defined as $\tau(x_0) = -\int_0^{\infty} dt\ t\ \frac{\partial \mathcal{S}_{x_0}(t,\mathcal{R})}{\partial t}$. A differential equation can be written and solved for $\tau(x)$, with absorbing ($\tau(b) = 0$) and reflecting ($\frac{\partial \tau}{\partial x}|_{x=a} = 0$) boundary conditions. The mean first passage time for a given position x equals,

$$\tau(x) = \frac{1}{D}\int_x^b dy\ e^{\frac{V(y)}{k_BT}} \int_a^y dz\ e^{\frac{-V(z)}{k_BT}}\ . \tag{S2}$$

Finally, the kinetic rate to unfold $k_{\rightarrow}$ is defined as the inverse of the mean first passage time evaluated at $a = x_{min}$ ($k_{\rightarrow} = \tau(a)^{-1}$),

$$k_{\rightarrow} = \frac{D}{\int_a^b dy\ e^{\frac{V(y)}{k_BT}} \int_a^y dz\ e^{\frac{-V(z)}{k_BT}}}\ . \tag{S3}$$

By equating Eq. (S3) and Eq. (1a) we get:

$$B = k_BT\left[\log\left(\frac{k_0}{D}\int_a^b dy\ e^{\frac{V(y)}{k_BT}} \int_a^y dz\ e^{\frac{-V(z)}{k_BT}}\right)\right]. \tag{S4}$$

For a DNA/RNA molecule forming a hairpin structure, the reaction coordinate x for the unfolding-folding reaction (N↔U) equals the number of released base-pairs m. As m is an

integer, the double integral in Eq. (S4) becomes a double sum with limits $a \equiv 0$ and $b \equiv M$. These limits correspond to the configurations where the molecule is folded in N ($m = 0$) and unfolded in U ($m = M$), respectively. Intermediate m values ($0 < m < M$) indicate partially folded configurations. Taking $dy, dz \equiv l_0$ equal to the inter-monomer distance, we can rewrite Eq. (S4) as,

$$B = k_B T \left[\log \left(\frac{k_0 l_0^2}{D} \sum_{m=0}^{M} \sum_{m'=0}^{m} e^{\frac{V_m - V_{m'}}{k_B T}} \right) \right] \tag{S5}$$

The diffusion coefficient satisfies $D \simeq k_0 l_0^2$, therefore we get Eq. (4).

S2 Reconstruction of the kinetic rates

To determine k_0, we use the fact that the kinetic barrier to unfold at zero force can be approximated by the folding free energy, i.e., $B_{ij}(f = 0) \simeq \Delta G_{ij}^0$. This result can be obtained from Eq. (4) by calculating $B_{ij}(0)$ for $\Delta G_m^0 = mg$ with $g > 0$ the average free energy per bp. Extrapolating $k_{i \to j}(f)$ to zero force, we can determine the attempt rate k_0 using Eq. (1a),

$$k_0 \simeq k_{i \to j}(f = 0) \exp \left(\frac{\Delta G_{ij}^0}{k_B T} \right) . \tag{S6}$$

To extrapolate the $k_{i \to j}(f)$ to zero force, we need to reconstruct the kinetic rates at sufficiently low forces. A possible strategy is to take advantage of the detailed balance condition by merging Eqs. (1b) and (2),

$$\log \left(\frac{k_{i \leftarrow j}(f)}{k_{i \to j}(f)} \right) = \frac{1}{k_B T} \left[\Delta G_{ij}^0 - \int_0^f (x_j(f') - x_i(f')) df' \right] , \tag{S7}$$

with x_i (x_j) being the extension of the initial (final) state, ΔG_{ij}^0 the folding free-energy, and $k_{i \to j}(f)$ ($k_{i \leftarrow j}(f)$) the force-dependent unfolding (folding) kinetic rate.

To illustrate and test the approach, we have analyzed the HI1 molecule by extrapolating the unfolding and folding kinetic rates to zero force. The extrapolation has been done by fitting simultaneously the unfolding kinetic rate to a quadratic function (in $\log k$ versus f scale) and imposing Eq. (S7) to reconstruct the folding rate. The term, $\int_0^f (x_j(f') - x_i(f'))df'$ in Eq. (S7) is determined by using the elastic parameters of ssDNA.[3] The extrapolated unfolding and folding kinetic rates of HI1 are shown as solid lines in Fig. S2 for $i, j = \{N,\ I,\ U\}$. Let us mention that the quadratic fits to the unfolding rates in a log-normal plot agree with the curvature reported in a previous study using a similar approach.[4] The

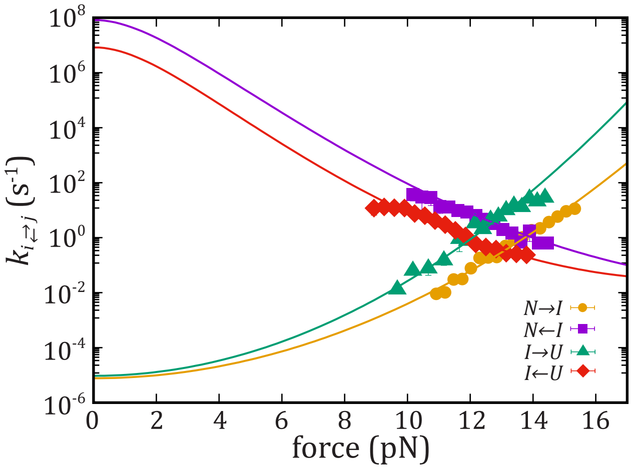

Figure S2: **Reconstructed kinetic rates.** Symbols correspond to the experimental values and solid lines correspond to the reconstructed kinetic rates.

extrapolated kinetic rate values from $k_{N\to I}(f)$ (yellow line in Fig. S2) and $k_{I\to N}(f)$ (green line in Fig. S2) are equal to $k_{N\to I}(0) = (7.7\pm1)\times10^{-6}s^{-1}$ and $k_{I\to N}(0) = (9.5\pm1)\times10^{-6}s^{-1}$. Using the average experimental folding free energy values reported in Tab. (1) and Eq. (S6), we get $k_0^{NI} = (8 \pm 1) \times 10^7 s^{-1}$ and $k_0^{IU} = (8 \pm 1) \times 10^6 s^{-1}$, which agree pretty well with the values summarized in Tab. (1) obtained by matching the experimental data to the predicted kinetic barriers (Eqs. (9a) and (9b)).

References

(1) Zwanzig, R. *Nonequilibrium statistical mechanics*; Oxford university press, 2001.

(2) Hyeon, C.; Thirumalai, D. Measuring the energy landscape roughness and the transition state location of biomolecules using single molecule mechanical unfolding experiments. *Journal of Physics: Condensed Matter* **2007**, *19*, 113101.

(3) Viader-Godoy, X.; Manosas, M.; Ritort, F. Sugar-Pucker Force-Induced Transition in Single-Stranded DNA. *International Journal of Molecular Sciences* **2021**, *22*.

(4) Rognoni, L.; Stigler, J.; Pelz, B.; Ylänne, J.; Rief, M. Dynamic force sensing of filamin revealed in single-molecule experiments. *Proceedings of the National Academy of Sciences* **2012**, *109*, 19679–19684.
